# When do dry cows get heat stressed? Correlations of rectal temperature, respiration rate, and performance

**DOI:** 10.3168/jdsc.2019-18019

**Published:** 2020-09-02

**Authors:** I.M. Toledo, T.F. Fabris, S. Tao, G.E. Dahl

**Affiliations:** 1 Institute of Food and Agricultural Sciences (IFAS) Extension, University of Florida, Gainesville 32603; 2 Department of Animal Sciences, University of Florida, Gainesville 32608; 3 Department of Animal and Dairy Science, University of Georgia, Tifton 31793

## Abstract

Previous studies have documented the negative effects of heat stress during the dry period on dairy cow performance during the next lactation, but an easy method to assess heat stress for dry cow management is lacking. In an effort to determine a threshold for heat stress based on the physiological response of respiration rate, an easily measured variable, an analysis including data collected from 6 different studies (n = 144 cows) was performed to summarize the correlations among rectal temperature (RT) and respiration rate (RR) during the dry period, milk production during the first 8 wk of lactation (MK), calf birth weight (CW), body weight at calving (BW), gestation length (GL), and dry matter intake (DMI) pre- and postpartum. Studies were conducted in Florida during the summer, and dry cows were assigned to 2 treatments: heat stress (only shade; HT, n = 75 cows) and cooling (shade, fans, and soakers; CL, n = 69 cows). Average RT (0.3°C) and RR [26 breaths per min (bpm)] of HT cows increased compared with CL. In addition, the CL group produced more milk (2.8 kg/d) during the first 8 wk of the subsequent lactation relative to HT. Correlation analysis indicated that RR of HT was negatively correlated with MK and GL. Within HT, RT was also negatively correlated with MK and GL and tended to be negatively correlated with CW. In addition to being negatively correlated with RT and RR, HT MK was positively correlated with CW, GL, and DMI postpartum. In CL, no correlations were observed for RR, but RT was positively correlated with BW, and MK was positively correlated with DMI postpartum and tended to be positively correlated with GL. Moreover, the overall RT was 39.1 ± 0.48°C and RR was 61 ± 19.5 bpm, which indicates that RR over 61 bpm is an indicator of heat stress in cows during the dry period and can be easily assessed at the cow level. In summary, the more heat load a cow carries in the dry period, the greater the negative effects are on subsequent milk production, which may suggest a threshold for the effects of heat stress. Also, variation among cows within the HT group indicates that cows with longer gestation length have heavier calves, eat more postpartum, and produce more milk, but not as much as CL cows.

## Highlights

• The more heat load a cow carries in the dry period, the greater are the negative effects on subsequent lactation milk production.
• Respiration rate measurements can be used as a non-invasive management tool to monitor and determine if dairy cows are heat stressed during the dry period.
• At 61 breaths per minute, cows are heat stressed.

## Graphical Abstract

**Figure:**
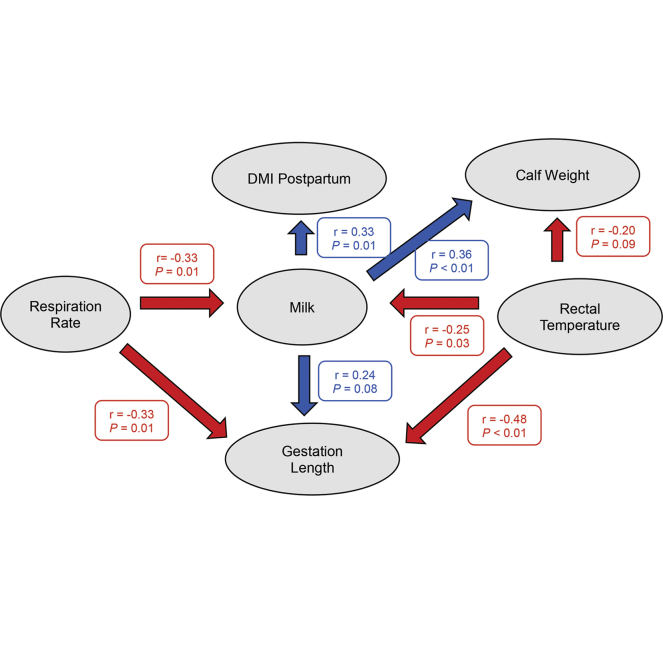
**Summary:** Heat stress during the dry period negatively affects dairy cow performance during the next lactation. Physiological measurements (respiration rate, rectal temperature) of cows during the dry period can be used to guide animal management and reduce negative outcomes of decreased milk production and smaller calves.

During the past 10 years, numerous studies have documented the negative effects of heat stress during the dry period on dairy cow performance during the next lactation. Exposure of cows to heat stress during the dry period is associated with compromised mammary cell proliferation when dry (Tao et al., 2011) and decreases in milk yield in the subsequent lactation (reviewed in Tao and Dahl, 2013). Reduced immune cell function during the transition period (do Amaral et al., 2010, 2011) and lower neutrophil response to intramammary infections during early lactation are also observed in cows exposed to heat stress during late gestation (Thompson et al., 2014). Moreover, greater incidence of postpartum disorders and lower reproductive performance are associated with seasonal exposure of cows to heat stress during the dry period (Thompson and Dahl, 2012). Dry period heat stress also decreases DMI and BW during the dry period, gestation length, and calf weight (do Amaral et al., 2011; Tao et al., 2011).

It has been demonstrated that lactating cows start to experience the effects of heat stress when the temperature humidity index is higher than 68 (Zimbelman et al., 2009). Although temperature humidity index is a useful tool to assess the degree of heat stress potential on the cow, her own physiological responses to heat should be a better indicator of her degree of heat stress. Increases in respiration rate and sweating are the primary means of dissipating excess heat under heat stress conditions. Subsequent increases in body temperature are indicators of the cow's inability to completely dissipate her heat load. Currently, no data are available showing a physiological threshold to identify that dry cows are on the verge of experiencing significant heat stress.

To better understand when dry cow heat stress affects performance, in the present study, correlation analysis of rectal temperature (**RT**) and respiration rate (**RR**) during the dry period, milk production during the first 8 wk of lactation, calf birth weight, cow body weight at calving, gestation length, and DMI pre- and postpartum were performed, by analyzing the data collected from 6 different studies conducted in Florida from 2007 to 2014. Our objective was to determine a threshold for RR that might be easily applied in dry cow management to predict the influence of heat stress on subsequent milk yield under typical management conditions.

All 6 studies were conducted at the University of Florida Dairy Unit (Hague, FL) during the summer months of 2007, 2008, 2009, 2010, 2015, and 2016. In all studies, animal handling and experimental procedures were approved by the Institutional Animal Care and Use Committee of the University of Florida. Approximately 46 d before expected calving, multiparous Holstein cows were dried off and randomly assigned based on mature-equivalent milk production of the previous lactation to one of 2 treatments: heat stress (**HT**, n = 75 cows) or cooling (**CL**, n = 69 cows). During the dry period, cows were housed in a freestall barn with a cooling system for CL cows consisting of shade, soakers, and fans, whereas HT cows were provided only shade. Fans ran continuously, whereas soakers turned on automatically for 1.5 min at 5-min intervals when the ambient temperature exceeded 22.2°C. After calving, cows from both treatments were housed in the same freestall barn and cooled by soakers and fans. Hobo Pro Series Temp probes (Onset Computer Corp., Pocasset, MA) were placed approximately in the center of each pen, under shade, and were used to measure air temperature and relative humidity every 15 min. We measured RT once daily (1430 h) during the dry period, using a GLA M700 digital thermometer (GLA Agricultural Electronics, San Luis Obispo, CA). Dry period RR was recorded thrice weekly (1500 h on Monday, Wednesday, and Friday) by counting flank movements for 1 min. Body weight at calving (BW) was recorded for each cow. Lactating cows were milked twice daily (0800 and 2000 h), and milk yield was recorded by the AfiLab milk analyzer (Kibbutz Afikim, Israel) at each milking. Dry cows were fed once a day (0800 h), and lactating cows were fed twice daily (0730 and 1300 h). Daily DMI was measured from dryoff until 42 d postpartum. Average daily milk production of the first 8 wk of lactation (**MK**) was included in the correlation analysis of the present study. Gestation length (**GL**) and calf birth weight (**CW**) were also recorded for cows in both groups.

We used PROC CORR of SAS University Edition (version 9.4, SAS Institute Inc., Cary, NC) to determine simple Pearson correlation coefficients. Correlations were calculated among RT and RR during the dry period, MK, CW, BW, GL, and DMI pre- and postpartum. Weekly averages for milk production, RT, and RR were analyzed using the PROC MIXED procedure of SAS. Weekly averages of RR and RT during the dry period and the weekly average for MK were used in the analysis. Cow nested within year × treatment was used as a random effect.

Consistent with the original reports (do Amaral et al., 2009, 2011; Tao et al., 2011; Thompson et al., 2014; Fabris et al., 2019), in the combined data set average (±SE) increases in RT (0.3 ± 0.03; *P* < 0.01; Table 1) and RR [26 ± 1.2 breaths per min (bpm); *P* < 0.01; Table 1] of HT cows were elevated compared with CL. In addition, in the present analysis, milk production of CL cows was 2.8 ± 0.8 kg greater (*P* = 0.01; Table 1) during the first 8 wk of the subsequent lactation relative to HT cows, which was expected from analysis of data from previous studies (do Amaral et al., 2009; Tao et al., 2011; Thompson et al., 2014).

**Table 1:** Rectal temperature (°C), respiration rate [breaths per min (bpm)] during the dry period and milk production during first 8 wk of lactation (kg/d) of cows exposed to either heat stress (n = 75) or cooling (n = 69) during the dry period (±SE)

Treatment	Heat stress	Cool	*P*-value
Rectal temperature (°C)	39.3 ± 0.03	38.9 ± 0.03	<0.01
Respiration rate (bpm)	74 ± 1.2	48 ± 1.2	<0.01
Milk production during first 8 wk of lactation (kg/d)	30.3 ± 0.8	33.1 ± 0.9	0.01

Correlation analysis indicated that RR and RT of HT cows were negatively correlated with MK (r = −0.33, *P* < 0.01, and r = −0.25, *P* = 0.03, respectively; Figure 1). Similarly, previous studies (do Amaral et al., 2011; Tao et al., 2011; Thompson et al., 2014) have shown that HT cows that have greater RT and RR than CL during the dry period have decreased milk production in the subsequent lactation. Moreover, it has been well documented that cows exposed to heat stress during late gestation have shorter gestation length and smaller calves compared with cows that are cooled during late gestation (Collier et al., 1982; do Amaral et al., 2011; Tao et al., 2011). In agreement, RR and RT of HT cows was negatively correlated with GL (r = −0.33, *P* = 0.01, and r = −0.48, *P* < 0.01, respectively; Figure 1). Moreover, RT of HT cows tended to be negatively correlated with CW (r = −0.20, *P* = 0.09; Figure 1).

**Figure 1:**
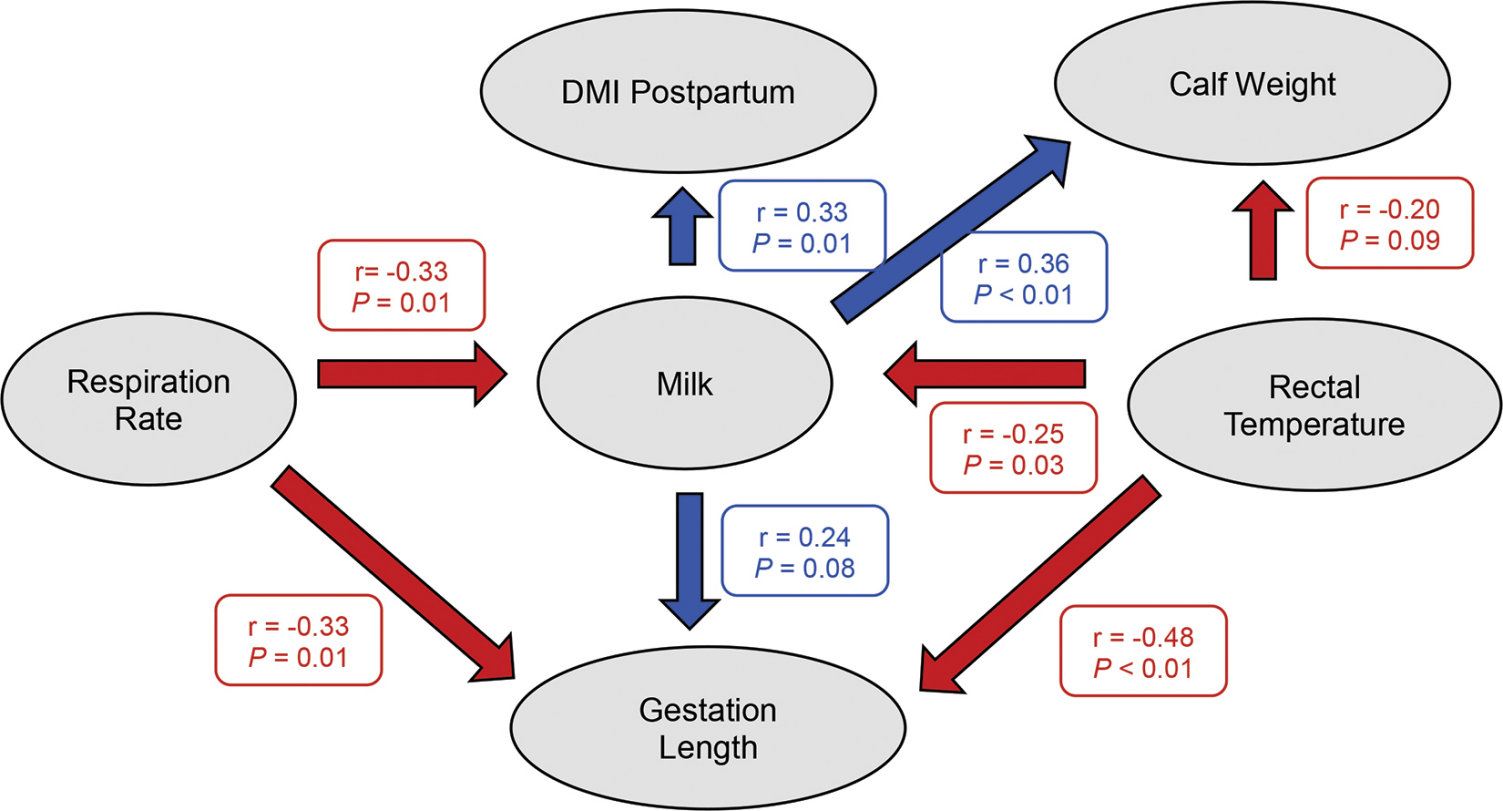
Simple correlation (r) analysis of data from heat-stressed cows indicated that respiration rates were negatively correlated with milk production during first 8 wk of lactation and gestation length. Rectal temperature was also negatively correlated with milk production during first 8 wk of lactation and gestation length and tended to be negatively correlated with calving weight. Milk production during first 8 wk of lactation was positively correlated with calving weight, gestation length, or DMI postpartum. Blue boxes and arrows represent positive correlations, and red boxes and arrows represent negative correlations.

Interestingly, in the present study, MK of HT cows was positively correlated with CW (r = 0.36, *P* < 0.01; Figure 1), GL (r = 0.28, *P* = 0.04; Figure 1), and DMI postpartum (r = 0.32, *P* = 0.01; Figure 1), indicating that the more heat load a cow carries in the dry period, the greater are the negative effects on milk production in the subsequent lactation, which may suggest a continuum for the effects of heat stress during the dry period. Thus, variation among cows within the HT group indicate that cows with longer gestation length have heavier calves, consume more DMI postpartum, and produce more milk; however, they do not produce as much milk as CL.

In CL cows, no correlations were observed between RR and BW, CW, MK, DMI, or GL, but RT was positively correlated with BW (r = 0.25, *P* = 0.03; Figure 2), and MK was positively correlated with DMI postpartum (r = 0.33, *P* = 0.01; Figure 2) and tended to be positively correlated with GL (r = 0.24, *P* = 0.08; Figure 2), indicating the positive effect of cooling during the dry period on gestation length, DMI postpartum, and milk production in the subsequent lactation. Indeed, even with effective cooling systems, cows will experience heat stress and have increases in RT during times of high ambient temperatures in a day, and those fluctuations in RT may affect future productivity.

**Figure 2:**
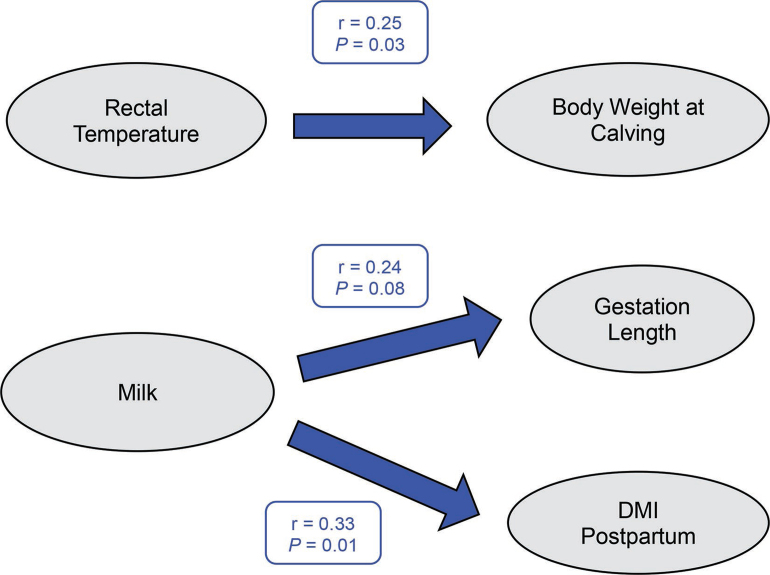
Simple correlation (r) analysis of data from cooled cows indicated that rectal temperature was positively correlated with body weight at calving, and milk production during first 8 wk of lactation was positively correlated with gestation length and DMI postpartum. Blue boxes and arrows represent positive correlations.

In high-producing lactating cows, a linear relationship between increases in respiration rates and rectal temperatures has been observed (Zimbelman et al., 2009). In the present study, the analysis of 6 different dry-period heat stress experiments indicates that when combining RT and RR data from both CL and HT, the overall RT and RR averaged 39.1 ± 0.48°C and 61 ± 19.5 bpm, respectively. An RT of 39.1°C or higher is indicative of heat stress in cows, suggesting that RR over 61 bpm can be used as a non-invasive management tool to indicate heat stress in cows during the dry period.

In conclusion, results from the present study support the concept that the more heat load a cow carries in the dry period, the greater are the negative effects on milk production in the subsequent lactation, which suggests a continuum for the effects of heat stress during the dry period. In addition, the present findings indicate that respiration rate measurements can be used as a non-invasive management tool to monitor and determine whether dairy cows are heat stressed during the dry period.
